# Methodological choices in brucellosis burden of disease assessments: A systematic review

**DOI:** 10.1093/eurpub/ckac131.528

**Published:** 2022-10-25

**Authors:** C di Bari, N Venkateswaran, G Patterson, D Pigott, B Devleesschauwer

**Affiliations:** 1 Department of Epidemiology and Public Health, Sciensano, Brussels, Belgium; 2 Department of Health Metrics Sciences, Institute for Health Metrics and Evaluation, Seattle, USA; 3 Department of Population Medicine, University of Guelph, Guelph, Canada; 4 Department of Translational Physiology, Infectiology and Public Health, Ghent University, Ghent, Belgium; 5 Human Health, Global Burden of Animal Diseases, Liverpool, UK

## Abstract

**Background:**

Foodborne and zoonotic diseases such as brucellosis present many challenges to public health and economic welfare. Increasingly, researchers and public health institutes use disability-adjusted life years (DALYs) to generate a comprehensive comparison of the population health impact of these conditions. DALY calculations entail several methodological choices and assumptions, with data gaps and uncertainties to accommodate. The following review identifies existing brucellosis burden studies and analyses their methodological choices and assumptions.

**Methods/Findings:**

A systematic search for brucellosis burden calculations was conducted in pre-selected international and grey literature databases. Using a standardized reporting framework, we evaluated each estimate on a variety of key methodological assumptions necessary to compute a DALY. One study reported estimates at the global level, the rest (13) at national or subnational. Most studies retrieved brucellosis epidemiological data from administrative registries. Incidence data were often estimated based on laboratory-confirmed tests. Not all studies included mortality estimates (YLLs) in their assessments due to the lack of data or the assumption that brucellosis is not a fatal disease. Only two studies used a model with variable health states and corresponding disability weights. The rest used a simplified singular health state approach. Wide variation was seen in the duration chosen for brucellosis, ranging from 2 weeks to 4.5 years, irrespective of whether a chronic state was included.

**Conclusions:**

Available brucellosis burden assessments vary widely in their methodology and assumptions. Further research is needed to characterize better the total clinical course of brucellosis and estimate case-fatality rate. In addition, reporting of methodological choices should be improved to enhance transparency and comparability of estimates. These steps will increase the value of these estimates for policymakers.

**Key messages:**

• Inconsistencies in reporting methods and assumptions are found, which hinder transparency and understanding of the methodological choices and the reuse of estimates for prioritization purposes.

• Thus, there is a need for a more standardized reporting system for DALY estimates, which could resemble a checklist that reports the methodological choices and assumptions.

